# The last imprint: Differential methylation assay for forensic identification of brain tissue and death determination

**DOI:** 10.14440/jbm.0231

**Published:** 2025-11-14

**Authors:** Gal Bilinsky, Dan Grinstein, Anat Zvi, Adi Beth-Din, Ofir Israeli

**Affiliations:** 1 Department of Biochemistry and Molecular Genetics, Israel Institute for Biological Research, Ness Ziona, Central District 74100, Israel; 2 Science & Technology Unit, Ministry of Defense, Hakirya, Tel Aviv 6473424, Israel

**Keywords:** Bisulfite sequencing, Brain, Crime scene, DNA methylation, Genomics, High-throughput sequencing

## Abstract

**Background:**

The ability to test samples for the presence of specific tissues is useful for numerous forensic applications. More specifically, the identification of vital organ remains, such as the brain, in a crime scene or battlefield, can assist in determining the death of a missing person. In many cases, tissue samples are of insufficient quality or quantity for the application of histological methods, leaving forensic labs mostly restricted to immunological and catalytic assays designed to identify blood, semen, and saliva. Recent studies have suggested expression profiling-based methods for tissue and bodily fluid identification.

**Objective:**

We present a methylation-based assay for the detection of brain tissue in forensic samples.

**Methods:**

Genome-wide methylation data from 12 human tissues were analyzed to identify CpG sites uniquely methylated in brain tissue. Four candidate regions were selected based on high inter-tissue specificity and low intra-tissue variability. Targeted assays were developed using bisulfite conversion, polymerase chain reaction amplification, and next-generation sequencing, and validated based on reference tissues, mixtures, and environmentally degraded DNA samples.

**Results:**

Four regions displayed consistent brain-specific methylation with >94% single-read accuracy and complete sample-level discrimination at ≥5% brain DNA. The assay retained diagnostic performance in mixed and degraded samples, demonstrating robustness under typical forensic conditions.

**Conclusion:**

This study presents a sensitive and specific methylation-based assay for brain tissue identification. The approach enables reliable detection in degraded or composite materials and supports future integration of epigenetic biomarkers into forensic workflows for organ-source attribution.

## 1. Introduction

Molecular-based differential diagnosis of tissues is paramount in forensic science, facilitating precise identification of human remains in crime scenes or battlefields. Such assays provide useful information for police investigations, such as the determination of cause and manner of death.^1^ The molecular approach utilizes specific molecular markers, such as DNA methylation patterns or protein expression, to distinguish between tissues that may exhibit similar macroscopic or microscopic features.^2^ The application of molecular techniques is particularly invaluable in cases involving decomposed or fragmented remains, where traditional methods such as visual inspection or histology may come short. By examining unique molecular signatures, forensic scientists can overcome challenges posed by degraded samples and ambiguous anatomical features, ensuring reliable identification and precise determination of tissue types. Molecular-based differential diagnosis represents a transformative advancement in forensic science, enhancing traditional methodologies with robust molecular tools to resolve complex cases with greater accuracy and reliability. These tools underscore the pivotal role of molecular biology in forensic investigations.

When small amounts of human tissues are found in disaster, war, or crime scenes, forensic scientists can employ DNA analysis to confirm the identity of an individual, linking the tissue to a specific missing person.^3^ However, determining the identity of a missing person is not always sufficient to establish their status or whether the person is dead or alive, particularly in cases where a corpse or major remains are not found. Detection of brain remains, particularly the cerebral cortex, might be instrumental in aiding the determination of death in missing person cases. Although the presence of brain matter indicates severe damage, it may be necessary to provide other evidence before drawing the conclusion that this indicates death rather than serious injury. The cerebral cortex, the outermost layer of the brain, is responsible for higher-order functions such as cognition, perception, and voluntary motor control.^4^ The detection of cortical tissue at a crime scene can provide evidence of life cessation, as the cortex is essential. Due to its proximity to the skull, the cortical tissue is more likely to be affected in acute injuries such as gunshot wounds or explosions compared to deeper brain structures. From a medico-legal perspective, the identification of brain remains significant for the determination of death and the legal resolution of a missing person’s case.^5^ The determination of a missing person’s fate as quickly as possible is important for rescue efforts, as well as for the victim’s family.

Classic histopathological methods, while invaluable in clinical and some forensic contexts, may not be adequate for analyzing brain which remains from war, disaster zones, or crime scenes due to several limitations. Histopathology involves the microscopic examination of tissue sections to study the manifestations of disease, necessitating well-preserved tissue samples. This is often not the case in scenarios involving severe trauma, extensive decomposition, or exposure to harsh environmental conditions typical of war or crime scenes.^6^ In such settings, brain tissue is frequently fragmented, degraded, or contaminated, rendering traditional histopathological techniques ineffective. Histopathological methods rely on the structural integrity of cells and tissues, which can be severely compromised over time, especially under adverse conditions, hampering the ability to obtain clear, interpretable histological slides.^7^ Furthermore, the presence of environmental contaminants, such as soil, other tissues, and microorganisms, can obscure cellular details and interfere with staining procedures used in histopathology.^8^

To address these challenges, molecular-based methods offer a robust and effective alternative. Techniques such as DNA analysis, RNA sequencing, and protein-based assays might provide critical information even from highly degraded samples. DNA analysis has been proven effective in identifying individuals from minimal and compromised biological material, as it can be extracted from small fragments of tissue and withstands decomposition better than cellular structures.^3^ Certain mutations, structural changes, or variances in mitochondrial DNA could help identify a tissue to some extent; yet, DNA sequencing cannot distinguish tissue types, as all cells generally contain an identical genetic sequence. Hence, molecular tissue detection methods use either protein markers, which are uniquely expressed in the target tissue, or rely on the epigenetic cellular machinery, such as mRNA markers and DNA methylation. While RNA species such as mRNA and microRNA hold promise for tissue-specific diagnosis, mRNA molecules are susceptible to environmental degradation, primarily due to RNAse activity, limiting their utility in field conditions. MicroRNA molecules, while exhibiting greater stability, are characterized by a limited repertoire. Given the need for a robust, stable molecular marker that retains tissue-specificity under variable environmental conditions, DNA methylation emerged as a suitable alternative. Another important advantage of DNA methylation is that completely distinct patterns are expected for different tissues, as opposed to mRNA species, which exhibit differential expression levels. This may enable a more accurate detection in mixed samples by reducing the need for complex signal deconvolution. For those considerations, DNA methylation profiles were employed in the assay presented in this study. DNA methylation serves as a pivotal epigenetic mechanism involved in modulating gene expression. Within the brain, epigenetic processes control gene expression relevant for neurobiological and cognitive pathways. Various methylation databases house information regarding tissue-specific methylation patterns.^9^-^11^ Moreover, methylation, being a robust genetic modification, exhibits remarkable stability across diverse environmental conditions. Jones *et al.*,^12^ for example, have demonstrated that storage of blood samples at room temperature for over a year does not affect their methylation levels. Although DNA methylation is generally stable, it can be impacted by storage techniques, environmental factors, and DNA extraction procedures.

A well-established method for detecting methylated DNA relies on bisulfite conversion, offering sensitivity and specificity.^13^ Bisulfite treatment efficiently converts unmethylated cytosine bases to uracil while leaving methylated nucleotides intact. Polymerase chain reaction (PCR)-based assays utilizing designed primers and probes allow the detection of methylation abundance at specific sites. Alternatively, more precise diagnoses can be achieved through high-throughput sequencing (HTS) of specific regions. This allows the observation of many linked sites in a single molecule, which provides greater accuracy, diminishing the likelihood of false positive results from either technical or biological sources. For instance, Thompson *et al*.^14^ identified discrete genomic loci within beta-cell tissue characterized by exceptionally low methylation levels. While individual CpG sites within these loci exhibited a relatively high degree of methylation variability across different tissue types (approximately 20% unmethylated in other samples), the unique combination of demethylated CpG sites within these regions served as a distinctive epigenetic marker, enabling the precise differentiation of beta cells from other cell types.^14^

Recent studies have investigated the unique pattern of methylation in the brain. Braun *et al*.^15^ examined general methylation patterns in different cell types and found distinct methylation patterns prominently in brain tissue, both in neuronal cells and non-neuronal cells, compared to various other tissues such as blood or muscle. Another recent study further delineates the methylation profile in two regions of the brain, the cortex and the cerebellum, compared to samples such as blood, revealing distinctly different profiles in each tissue type.^11^,^16^,^17^

In this study, we present a comprehensive methodology for the differential identification of brain tissue in forensic samples. Our approach included collecting reduced representation bisulfite sequencing results deposited by various studies, mapping methylation profiles in human brain and control tissues, and predicting the best genomic areas for tissue-specific differential analysis assays. Variable brain samples were used, including samples from Down syndrome-affected individuals. The inclusion of samples from individuals with Down syndrome was to make sure the final markers reflected universally conserved basic regulatory functions. Naturally, markers that are common across the population are desired for forensic applications. In addition, CpG sites that are expected to be specifically unmethylated in brain samples were obtained from Yuval Dor’s lab.^11^,^18^,^19^ Dor’s lab applied a different approach, utilizing chromatin immunoprecipitation sequencing technology to find single CpG sites exhibiting unique hypo-methylation in multiple sorted brain cell types, with single methylation sites unique for neurons and astrocyte cells.

This paper outlines the development of a novel analytical method, employing bisulfite treatment, targeted amplification, and HTS, to reliably detect brain tissue within samples that have undergone conditions such as burning, sun-drying, or mixing of biological material from different tissues.

## 2. Materials and methods

### 2.1. Materials

Table 1 contains information about biological samples, software, equipment, and the various reagents and consumables used.

**Table 1:** Materials and instrumentation used in this study

Type	Materials/Instruments	Source
Biological samples	Human tissue samples (brain cortex, skin, muscle, artery, stomach, bone marrow, liver, and heart atrium)	Amsbio (UK)
	Blood samples	Magen David Adom (Israel)
Consumables	AMPure XP Beads	Beckman Coulter (USA)
	HT1 buffer	Illumina (USA)
	PhiX Control V2 library	
	Zymo-Spin™ columns	Zymo Research (USA)
Enzymes and buffers	Nextera XT Index Kit (N7×x and S5×x primers)	Illumina (USA)
	RNAse	Qiagen (Germany)
	ATL buffer and proteinase K	
	Multiplex PCR mix and Q solution	
	Kapa HIFI ready mix	Roche (Switzerland)
	CT conversion reagent, M-binding buffer, M-wash buffer, L-desulphonation buffer, M-elution buffer	Zymo Research (USA)
Equipment	TapeStation	Agilent (USA)
	MiSeq system	Illumina (USA)
	PCR machine	Qiagen (Germany)
	QIAcube robot	
Software	Bisulfite-primer-seeker	Zymo-Research, available from: https://zymoresearch.eu/pages/bisulfite-primer-seeker (version N/A)
	Bismark	Babraham Bioinformatics (version 0.22.3, UK)
	Bowtie2	Johns Hopkins University (version 2.2.0, USA)
	Matplotlib	Open source (version 3.5.3)
	Seaborn	Open source (version 0.12.2)
	Scikit-learn	Open source (version 0.23.2)

Abbreviations: PCR: Polymerase chain reaction; UK: United Kingdom; USA: United States of America.

### 2.2. Selection of potential diagnostic genomic loci

Genomic methylation sequencing data were collected from the following sources: seven brain samples and 24 other adult human tissues, deposited by the NIH Roadmap Epigenomics Mapping Consortium,^1^ as well as four additional frontal cortex brain samples from healthy individuals and individuals with Down syndrome^16^ (Sequence Read Archive [SRA] accession: SRR3536980, SRR3537006, SRR3537007, and SRR3537016). Some of the samples were obtained as “. bed” files, which contain methylation percentage for each covered base in the human genome assembly reference (GRCh37),^20^ while other samples consisted of raw FASTQ files and required preliminary processing. These FASTQ files were aligned to the human genome using Bismark^21^ and bowtie2.^22^ The positional information as “.bed” files for all samples was extracted using the “bismark_methylation_extractor” and “bismark2bedGraph” modules. A ”hypo,” “hyper,” and “informativeness” score was calculated for each covered position in the human genome, using an in-house python script, depicting how close the position is to being completely unmethylated in all brain samples and completely methylated in all other samples, in the case of hypo-methylated regions, or vice versa in the case of hyper-methylated regions. Major exceptions were penalized logistically (Equation [S1]). Subsequently, potential genomic regions were ranked by the following scheme: regions were required to contain five or more CpG methylation sites within a span of 300 bp and were scored by the number of CpG points and the average “informativeness” score of the 50% percentile best points in the region (Equation [S2]). Accordingly, 11 top-ranking, potential genomic regions were selected. Two additional regions were obtained from the results of Kohn *et al*.^18^ and Lubotzky *et al*.^19^ One assay was predicted in both. To rule out the possibility of animal tissue affecting results, we used BLAST to make sure no genomes, except human, which contain the sequence of the expected PCR product, or similar sequences with >95% identity.

### 2.3. Collection of tissue samples

Fresh-frozen human tissues, obtained shortly after death or promptly preserved post-surgery, were purchased from Amsbio (United Kingdom; see more details at https://www.amsbio.com/products/biorepository/tissues). These samples, delivered in a frozen state, were all derived from non-cancerous, normal tissue sources, collected within a post-mortem interval ranging from 3 to 9 h (Table 2). All tissue specimens were collected under ethical regulations and in accordance with all applicable (local and international) laws, with appropriate Institutional Review Board/Independent Ethics Committee approval. All donations were made under a voluntarily signed Informed Consent form. All specimens were delivered de-identified and de-linked from the original clinical records by Amsbio. The blood sample was obtained from the Israeli National Blood Bank of Magen David Adom in Tel HaShomer, Israel.

**Table 2:** Human tissue samples used in this study

Specimen ID	Anatomical site	Sample name	Gender	Age	Cause of death	Procurement date	PMI (h)
057-105	Skin	Skin	Female	55	Healthy volunteer	03/01/2018	NA
46-35723	Striated muscle	Muscle	Male	60	Cardiac fraction	04/14/2015	5
113-T2789	Brain cortex	Brain	Male	65	Cancer; normal tissue recovery	12/01/2018	9
90-06-11 A	Endothelium, artery	Artery	Male	55	Acute heart failure	10/16/2014	5
A-1 N1	Stomach	Stomach	Female	70	Cardiovascular insufficiency	12/10/2008	7
46-40090	Liver	Liver	Male	71	Brain edema	08/17/2018	7
B-22	Heart, atrium	Heart	Male	48	Car accident	01/07/2007	3
46-41097	Bone marrow	Bone marrow	Male	53	Cirrhosis of the liver	04/21/2020	6
531132A	Brain cortex	Brain 2	Male	65	Pulmonary embolism	06/10/2018	9
531144A	Brain cortex	Brain 3	Male	65	Acute myocarditis	07/03/2018	9
	Whole blood	Blood	NA	NA	Live donor	05/05/2022	NA

Abbreviations: NA: Not applicable; PMI: Post mortem interval.

### 2.4. Crime scene or battlefield simulation and mixed samples

Brain, skin, and muscle tissue samples (50 mg each) were left in the field for 1 or 3 days in open test tubes placed inside a transparent box covered with a net. During the exposure period (May 24–27, 2023), ambient temperatures ranged from 18°C to 29°C with relative humidity between 50% and 75%, under clear, dry conditions typical of the eastern Mediterranean climate in late spring. For each sample, a replicate was first burned using a small butane flame for three seconds, while the tissue was placed on a glass microscope slide. The open flame was applied directly to the tissue surface, causing pronounced charring without ignition. In addition, 100 mg of brain cortex (113-T2789), heart, muscle, and skin samples were mixed to obtain mixed samples of 10% or 50% brain samples by weight (weighted and mixed as solid tissue before DNA extraction).

### 2.5. DNA extraction and bisulfite treatment

Each tissue specimen, weighing 25 mg, underwent manual sectioning into thin strips using a scalpel. Subsequently, 2 mg of each tissue was transferred to a tube for vigorous pipetting of 180 μL ATL buffer and 20 μL proteinase K (Qiagen, Germany) with the tissue sample, followed by incubation at 56°C and agitation at 200 RPM until complete dissolution. RNAse treatment was applied, and the samples were transferred for further processing using the tissue sub-protocol on the QIAcube robot (Qiagen, Germany). The blood sample extraction procedure was conducted on the QIAcube robot using the DNA blood mini protocol, adhering to established protocols (Qiagen, Germany). DNA quality assessment was performed using the TapeStation device (Agilent, United States) with the genomic DNA tape. The degree of DNA fragmentation was quantified in terms of DNA integrity number (DIN), where a higher score indicates less fragmentation. Intact, high-molecular-weight DNA typically yields a DIN score approaching 10, while extensively fragmented DNA scores closer to 1.

The bisulfite treatment was performed using reagents from ZymoResearch’s EZ DNA methylation-lightning kit. A total of 130 μL of CT conversion reagent was added to 20 μL of DNA (optimal DNA amount: 200–500 ng). After thorough mixing, the mixture underwent hybridization at 98°C for 8 min, followed by 54°C for an hour. Subsequently, 600 μL of M-binding buffer was combined with the previous mixture on Zymo-Spin™ columns, agitated, and centrifuged (>10,000 × g) for 30 s. Then, 100 μL of buffer M-wash was added and centrifuged, followed by the addition of 200 μL of buffer L-desulphonation. After a 15–20-min incubation at room temperature, the mixture was centrifuged again, and 200 μL of buffer M-wash was added and centrifuged two more times. The columns were placed in 1.5 mL test tubes, and 12 μL of buffer M-elution was directly added to the column. Final centrifugation for 30 seconds at full speed was performed to elute the DNA.

### 2.6. Primer design and HTS

#### 2.6.1. Amplicon library preparation for sequencing

Primers were designed using bisulfite-primer-seeker software^23^ with default parameters allowing for one degeneration per primer. Primers are listed in Table 3.

**Table 3:** Assay primers

No.	Forward primer	Reverse primer	Type	Origin	Coordinates (hg19.p13)		

					Chromosome	Start	End
n1	(TCGTCGGCAGCGTCAGATGTGTATAAGAGACAG) TATTATGTTATGGGAGGTTGTTTYGAG	(GTCTCGTGGGCTCGGAGATGTGTATAAGAGACAG) ACRTAAAACTTAAAACTCTTACAAATACT	Hypo	This paper	10	3,283,768	3,284,144
n2	(TCGTCGGCAGCGTCAGATGTGTATAAGAGACAG) TAAAAGGGAAATTGGATTTTTAGAGAGA	(GTCTCGTGGGCTCGGAGATGTGTATAAGAGACAG) ATCCATCCATTTTAATAATAAACACCAC	Hypo	This paper	9	7,209,139	7,209,478
n3	(TCGTCGGCAGCGTCAGATGTGTATAAGAGACAG) TGTGTTTGGTTTTTTAATAAAGGAAAGG	(GTCTCGTGGGCTCGGAGATGTGTATAAGAGACAG) CTTAAACTTCCCCTTCTCCTTCTC	Hypo	This paper	16	88,677,634	88,677,983
n4	(TCGTCGGCAGCGTCAGATGTGTATAAGAGACAG) TTTTTTTTGTTGGTAGTTGGAGGTAG	(GTCTCGTGGGCTCGGAGATGTGTATAAGAGACAG) CACAAAATAACAAATAACCTTACTCCCT	Hypo	This paper	6	112,086,098	112,086,366
n5	(TCGTCGGCAGCGTCAGATGTGTATAAGAGACAG) AGGTATGAAAGGTTAGGTTGTTTTTTTA	(GTCTCGTGGGCTCGGAGATGTGTATAAGAGACAG) CTCCTTTTCCCATTTTAAACACAATTAT	Hypo	This paper	6	128,387,907	128,388,072
n6	(TCGTCGGCAGCGTCAGATGTGTATAAGAGACAG) TAGATGAATTTGTAAAAGGGAAATTGGA	(GTCTCGTGGGCTCGGAGATGTGTATAAGAGACAG) CATCCATTTTAATAATAAACACCACACC	Hypo	This paper	9	7,209,139	7,209,478
n7	(TCGTCGGCAGCGTCAGATGTGTATAAGAGACAG) TAGGAGGTTTTGGCGTTCGGTTAGTTTT	(GTCTCGTGGGCTCGGAGATGTGTATAAGAGACAG) ACCAAAAAAAACTCCTACTACTAAAACA	Hyper	This paper	14	70,039,000	70,039,294
n8	(TCGTCGGCAGCGTCAGATGTGTATAAGAGACAG) AATTGGTAGGTTTGTAGTAGGAGGT	(GTCTCGTGGGCTCGGAGATGTGTATAAGAGACAG) TACAAAATAAACTAAAACTATTCCACRC	Hyper	This paper	17	72,353,358	72,353,665
n9	(TCGTCGGCAGCGTCAGATGTGTATAAGAGACAG) TTGTTTTTTAGTTTTGTAYGTTTTTTTT	(GTCTCGTGGGCTCGGAGATGTGTATAAGAGACAG) TCRTAAATTCAATACCATTAATAACCAA	Hypo	This paper	1	568,193	568,528
n10	(TCGTCGGCAGCGTCAGATGTGTATAAGAGACAG) AGGGAAGGGAATTTTAATTGTTTTTTTT	(GTCTCGTGGGCTCGGAGATGTGTATAAGAGACAG) ACTTAAAAACATATTTTAAATTTTTATCCCTTACA	Hypo	This paper	2	201,321,569	201,321,769
n11	(TCGTCGGCAGCGTCAGATGTGTATAAGAGACAG) ATTTGATTTTGTGGTAGTGGA	(GTCTCGTGGGCTCGGAGATGTGTATAAGAGACAG) AAAATCCCCACCTCTACTTAA	Hypo	Dor’s lab	16	72,460,052	72,460,163
n12	(TCGTCGGCAGCGTCAGATGTGTATAAGAGACAG) TATATGTGTGTAGGTTGAATAAAAT	(GTCTCGTGGGCTCGGAGATGTGTATAAGAGACAG) TCCATTTCATATCAATACTAATATT	Hypo	Dor’s lab	10	3,283,855	3,283,972
n13	(TCGTCGGCAGCGTCAGATGTGTATAAGAGACAG) TTTAGTGTTAGAATTGAAAGAGTAGA	(GTCTCGTGGGCTCGGAGATGTGTATAAGAGACAG) TTAACCTTAACTATATCTAACAAAAA	Hypo	This paper	1	10,239,929	10,240,207

Note: The table contains information about potential assay genomic loci, including primers designed (which includes the adapter overhang in parentheses), locus type (whether it is expected to be hypo-methylated or hyper-methylated in brain tissue), and origin (two areas were predicted by Krueger and Andrews,^21^ and Langmead and Salzberg^22^).

#### 2.6.2. Dual amplification library preparation

The library preparation followed the 16S metagenomics sequencing library preparation protocol (Illumina, United States) with a few modifications. Bisulfite-treated DNA served as the template for library amplification. The process included two amplification stages. In the first stage, segments were amplified using primers constructed from a specific part at the 3’ end suitable for sequences in the diagnostic test areas and universal sequences at the 5’ end complementary to sequences on the MiSeq flow cell:

(i) Forward: 5’ TCGTCGGCAGCGTCAGATGTGTATA AGAGACAG+ (Diagnostic bisulfite-specific sequence)
(ii) Reverse: 5’ GTCTCGTGGGCTCGGAGATGTGTATA AGAGACAG+ (Diagnostic bisulfite-specific sequence).

To each reaction tube, six μL of Multiplex PCR mix (Qiagen, Germany), 0.5 μL of Q solution (Qiagen, Germany), 1 μL of DNA from the bisulfite reaction, and 1.25 μL of each primer forward/reverse at a concentration of 1 pmol/μL. The samples were transferred for amplification in a PCR machine using the program: 95°C for 15 min, 40 cycles of 94°C for 0.5 min, 58°C for 1.5 min, 72°C for a min, and one cycle of 60°C for 5 min.

The DNA library underwent cleanup and size sorting at a 1:1 ratio using the AMPure XP beads. The purified product was eluted in 20 μL ddH_2_O.

In the second amplification, we used universal primers P5 and P7 from the Nextera XT index kit (Illumina, United States), which also contained an eight-base barcode to allow the libraries to run in multiplex. Before sequencing, the libraries were normalized, pooled, and quantified for multiplex running. The second amplification was performed with a reaction mixture that included 15 μL of the purified product from the previous stage, 25 μL of Kapa HIFI ready mix (Roche, Switzerland), and 5 μL of each primer of the second amplification, which contained a barcode and Illumina recognition sequences: Nextera XT index kit1 (N7xx) and Nextera XT index kit2 (S5xx).

The samples were placed in a PCR thermocycler and subjected to the following program: an initial denaturation at 95°C for 3 min, followed by eight cycles of 95°C for 30 s, 55°C for 30 s, and 72°C for 30 s, and a final extension at 55°C for 5 min.

In the final stage, another purification was conducted with AMPure XP beads, similar to the purification described in the previous stage. The purified product was eluted in 20 μL ddH_2_O.

#### 2.6.3. Quantification, quality testing, and sequencing on the MiSeq device

Amplicon library quantification and quality assessment were conducted on the Agilent TapeStation in the HS DNA application. Libraries were normalized to 2.5 nM, denatured with 0.2 N sodium hydroxide, and diluted 1:100 in HT1 buffer (Illumina, United States). Sequencing was performed on the MiSeq device (Illumina, United States) using the V2 kit with paired-end reads of 150 bases. A PhiX control V2 library (Illumina, United States) at a final concentration of 5% served as a positive control in each run. All sequence files are deposited in the NCBI SRA (accession# PRJNA1162678).

### 2.7. Bioinformatic analysis of sequencing data

FASTQ files yielded from HTS were analyzed using an in-house Python script. Reads were aligned to relevant reference sequences, and filtered by the following criteria: (i) Alignment identity > 85%, (excluding cytosines in CpG points [any CG in sequence]); (ii) the primer sequence was identical to the expected primer sequence; and (iii) all CpG points in the region were properly covered by the read and contain either CG or CT. Each valid read was then saved, and the specific methylation pattern and methylation percent were kept and counted. A sample was discarded if it contained fewer than 100 valid reads. The distribution of methylation percentage for each region was found and presented using the matplotlib^24^ and seaborn^25^ Python modules. Lines were smoothed for presentation using the kernel density estimate option in seaborn. The methylation distribution of all samples was compared, and a simple decision tree model of limited depth was employed to predict the origin of each read in a sample using scikit-learn.^26^ Input parameters for each model were the methylation state of each CpG position in a read, and the output label was a binary brain/non-brain result. Twenty percentages of the read data were reserved as a test set, not used in training, which allowed the extraction of reliable precision and recall values for the model generated for each assay. These values were used to assess the informativeness of each assay for tissue classification. The structure of model trees was examined to check if complex methylation patterns allow for better results than a simple linear regression model based on methylation percentage alone.

Once it was determined that complex methylation patterns did not improve accuracy, sample diagnostics were performed using the plotting script described above. The distribution of methylation percentages in reads from the unknown sample and controls was examined. The script also calculates and prints the fraction of reads with a given min/max methylation percentage in samples and controls.

## 3. Results

### 3.1. Prediction of potential diagnostic regions

**Figure 1:**
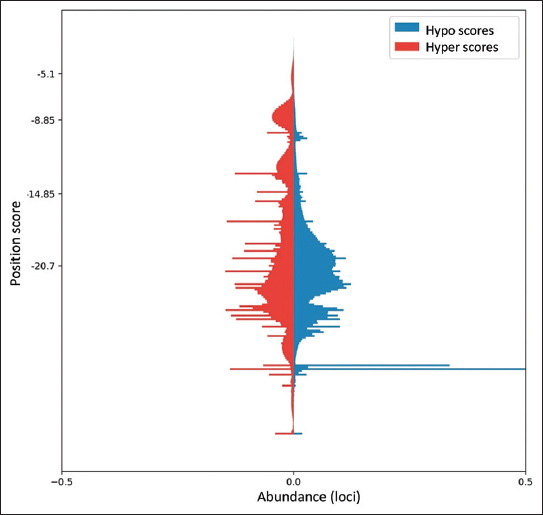
A histogram presenting the distribution of informativeness scores, “hypo”—under-methylated genomic positions in brain tissue (right, blue), and “hyper”—over-methylated genomic positions in brain tissue (left, red).

To identify potential loci along the genome that exhibit differential methylation profiles and could be utilized for diagnostics of brain tissue, we first collected data from documented genome-wide bisulfite HTS sequencing of 35 tissue samples (11 brain and 24 decoy tissues). These studies encompassed 74,987,111 possible CpG genomic positions along the human reference genome. This information was used to calculate a “hypo score” and “hyper score” for each position, reflecting the potential of each position to allow the distinctive identification of brain tissue as hypo-methylated or hyper-methylated, as compared to all other tissues. The score is formulated as a weighted distance between the ideal values and the actual values for a position, such that the highest possible score is 0. These scores are highest for genomic positions where all brain tissue samples are fully methylated and all other tissue samples are completely unmethylated, or vice versa. Approximately 0.003% of positions received a score >−2, with a score distribution showing an exponential behavior around this final range (Figure 1). A total of 13 potential genomic regions spanning 111–289 base pairs were chosen such that the score of CpG points contained in each region is maximized. These regions are predicted as likely to express a unique methylation profile in brain tissue samples. It is important to note that the data used do not allow the observation of complete sequences, but rather the average methylation rate at each specific point. This might limit the predictive power of the “hypo” and “hyper” scores, and thus, it is expected that only some of the tested loci will yield useful assays.

### 3.2. Differential diagnosis of hyper-methylated and hypo-methylated genomic regions

**Figure 2:**
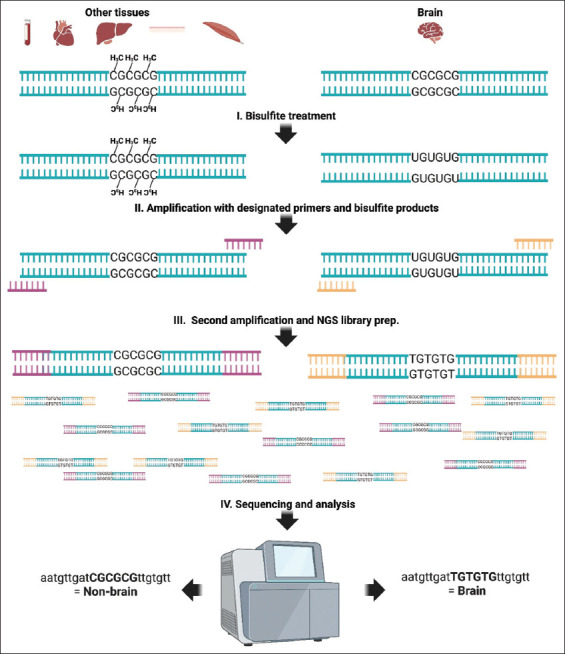
A schematic presentation of the established methodology for the differential diagnosis of hyper-methylated and hypo-methylated genomic regions. The illustration depicts a diagnostic genomic region that exhibits tissue-specific differential methylation patterns. Specifically, in this figure, this region is characterized by hypo-methylation in brain tissue, while demonstrating hyper-methylation in non-brain tissues. First is the bisulfite treatment of extracted DNA, which selectively converts unmethylated cytosine residues to uracil while leaving methylated cytosines (CCH3) unaltered. Second is first-step amplification, utilizing primers designed with a 3’ segment specific to the diagnostic regions and 5’ universal sequences complementary to Illumina flow cell adapters. In the second amplification step, each sample is assigned a unique molecular barcode to enable multiplex sequencing, and the samples are amplified for HTS libraries. Upon completion of the HTS process, the resulting sequence data undergoes analysis through a custom bioinformatic pipeline designed to quantify the presence of brain tissue-specific methylation patterns within the samples. Created in BioRender. Porat, T. (2025) https://BioRender.com/k74i920. Abbreviations: HTS: High-throughput sequencing; NGS: Next-generation sequencing.

The 13 potential diagnostic regions selected as described above served as a basis for designing an assay aimed at the differential identification of brain tissue in a forensic sample. To this end, we established a methodology based on bisulfite treatment, PCR amplification, and HTS of selected diagnostic regions. Following DNA purification, each tissue sample was treated with bisulfite conversion, a process that differentiates methylated and unmethylated cytosine bases. Methylated cytosines remain unchanged, while unmethylated cytosines are converted to uracil. Subsequently, we amplified targeted hyper-methylated and hypo-methylated genomic regions and combined this step with HTS library preparation. This involved two amplification phases. In the initial phase, specific DNA fragments were amplified using primers containing diagnostic region-specific sequences at the 3’ end and universal Illumina adapter sequences at the 5’ end. To enable sample multiplexing, each sample was uniquely barcoded (indicated in yellow and purple in Figure 2). These barcodes serve as molecular identifiers, allowing the pooling of multiple samples into a single sequencing run while maintaining sample-specific information. Overall, our methodology leverages the power of epigenome-wide association studies and differential DNA methylation analysis to identify tissue-specific methylation patterns, which can serve as biomarkers for various diagnostic applications, including the detection of brain tissue in burned and air-dried or mixed forensic tissue samples.

### 3.3. Diagnosis of the brain cortex using differential methylation

To assess the method’s suitability for molecular tissue characterization, we obtained fresh-frozen human tissues collected shortly after death or promptly preserved post-surgery. All tissues obtained were of normal, non-cancerous origin, harvested within a post-mortem window of up to 3–9 h. Ensuring sample freshness was crucial to maintain optimal tissue condition and uniform preservation quality, facilitating further potential testing under various environmental and preservation conditions. We opted for chemical shearing for the DNA extraction step due to consistently higher DIN values across all tissues, despite slightly lower DNA concentrations (5–6 ng/μL) as compared to mechanical shearing (data not shown). This concentration is adequate for subsequent bisulfite conversion. Initially, tissue samples of brain cortex, skin, blood, and muscle were prepared and sequenced (Figure 2) to evaluate the potential of different assays to distinguish between brain and other decoy tissues. The entire assay, including DNA extraction, bisulfite treatment, PCR reaction, HTS, and bioinformatic analysis, was performed for each region in each tissue. For each sequenced region, a simple decision tree model was trained to extract precision (reads correctly classified as brain tissue divided by all reads classified as brain tissue) and recall (reads correctly classified as brain tissue divided by all brain tissue reads) measures. By large, the precision measure was used to grade regions, as long as the recall value is not extremely small. This is because even if a small part of the brain reads were identified, the depth of HTS would still allow a successful identification for most samples. Meanwhile, low precision may indicate an inclination to false-positive results, which are extremely undesirable. The resulting models were also examined to test whether specific patterns of methylation states have better predictive power than the simple measure of methylation percent per read, revealing the existence of complex methylation patterns. No evidence of such better predictive power was observed. For this reason, diagnosis of samples can be just as accurate by simply requiring that a certain fraction of reads have a minimal/maximal methylation percentage (for hypo/hyper methylated regions) to classify it to contain brain tissue.

**Figure 3:**
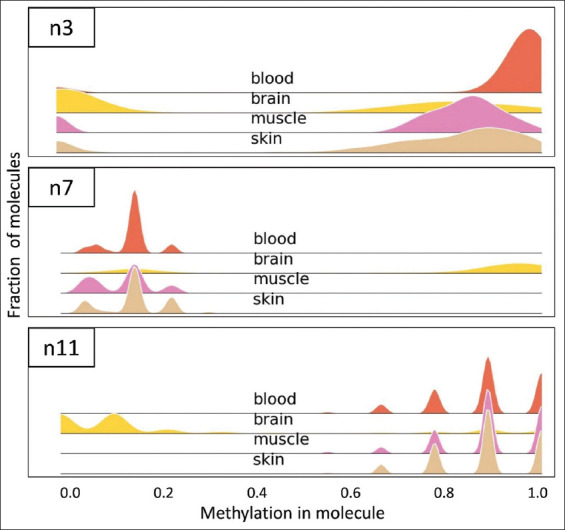
Histogram plots for three selected assays. The plots present the distribution of reads (each read representing a DNA molecule) by ranges of methylation % in CpG points for brain cortex, skin, muscle, and blood samples. Lines are smoothed for presentation using a kernel density estimate. Three assays were chosen to represent non-informative (n3), hyper-methylated (n7), and hypo-methylated (n11) assays. In the hyper-methylation assay (n7), brain tissue exclusively exhibits a “methylation in molecule” fraction of 0.8–1.0. Likewise, in the hypo-methylation assay (n11), brain tissue uniquely displays a “methylation in molecule” fraction of 0.0–0.2. Conversely, no tissue exclusively attains a low fraction of “methylation in molecule” in the non-informative assay (n3), which was designed originally as a hypo-methylated assay.

Of the 13 potential diagnostic regions tested in four tissue samples (brain, blood, muscle, and skin), five regions had the best potential to accurately identify brain tissue (Table 4): n1, n7, n8, n11, and n13. The diagnostic region n1 identified by our research group encompasses the diagnostic region previously reported by Dor’s laboratory n12. Consequently, we opted to proceed with the diagnostic regions n1. In those assays, a significant portion of the reads in brain samples showed a methylation profile that was absent in reads of other samples. For example, a hyper-methylated region in brain tissue was found at the loci amplified in assay n7 (Figure 3). In n7, about 60% of reads in the brain sample exhibited methylation percentage >90%, while almost all reads in other tissues presented methylation <40%, and none presented a profile identical to the unique brain profile. This is also reflected in the precision calculated for this assay (99%), showing high specificity (Table 4). Assay n11 is an example of an informative hypo-methylated region, at a slightly lower precision of 96%. Unlike those assays, assay n3, for example, does not seem to be informative. While the brain sample was somewhat hypo-methylated relative to the other samples, as predicted, there was no read profile completely unique to brain samples, which is reflected in a low precision value of 34%. It is important to note that these precision scores reflect the classification of a single molecule/read, and as sample results included 5 × 10^3^–5 × 10^6^ reads, actual sample verdicts are much more accurate. Thus, using reasonable thresholds should allow the detection of samples that contain <5% brain molecules. Each of the five best assays (n1, n7, n8, n11, and n13) allowed complete accuracy for this subset of tissue samples, when assuming a sample containing 10^5^ reads is composed of a random read subset of brain or decoy tissue, and thresholds are set to detect samples that contain 5% brain DNA or more. Complete sequence results for all tested loci are deposited in the SRA website (accession# PRJNA1162678).

**Table 4:** The 13 analyzed regions

Number	Length	Type	Precision (%)	Recall (%)	Number of CpG points
n1	289	Hypo	92	78	12
n2	208	Hypo	16	45	7
n3	267	Hypo	25	53	33
n4	187	Hypo	34	53	5
n5	138	Hypo	80	61	3
n6	217	Hypo	13	44	7
n7	268	Hyper	99	81	40
n8	204	Hyper	96	65	23
n9	224	Hypo	38	100	9
n10	201	Hypo	98	64	3
n11	111	Hypo	99	85	7
n12	117	Hypo	92	56	11
n13	210	Hypo	94	67	12

Notes: The table presents type and accuracy measures for 13 tested regions based around different genomic loci. Accuracy measures are derived from a simple decision tree model, trained to predict the origin of a single DNA read. Precision is defined as the fraction of reads classified as brain, which originate in brain samples. Recall is the fraction of reads in brain samples that are correctly classified as brain. The table is based on the initial sequencing of skin, brain, muscle, and blood samples.

### 3.4. Validation of selected assays in additional tissue samples

**Figure 4:**
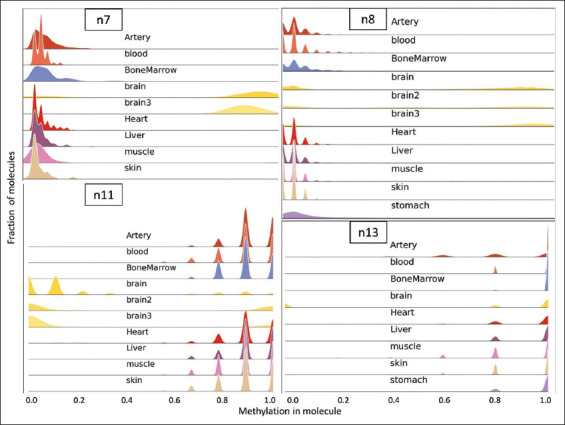
Histogram plots presenting the distribution of reads by ranges of methylation for assays n7, n8, n11, and n13. Notice the brain (in yellow) unique methylation pattern compared to all decoy tissues: in the hyper-methylation assays (n7 & n8), brain tissue exclusively exhibits a “methylation in molecule” fraction of 0.8–1.0. In the hypo-methylation assay (n11 & n13), brain tissue uniquely displays a “methylation in molecule” fraction of 0.0–0.2. Some tissue samples are omitted in cases where there was no successful amplification.

Five promising regions, presenting precision scores >94% in initial sequencing of four tissue samples and containing a dominant profile unique to brain tissue, were selected for further validation (regions n1, n7, n8, n11, and n13). First, these regions were tested using eight additional tissue samples: artery, stomach, bone marrow, liver, and heart atrium, as well as two additional brain cortex samples. Of these five regions, four presented methylation profiles that included a brain-unique pattern across the broader tissue panel: assays n7, n8, n11, and n13 showed near identical specificity for the new tissues tested, whereas assay n1 was omitted for further use. Liver tissue was found to contain some completely unmethylated DNA molecules at the n1 loci, leaving no profile unique to the brain. The resulting distributions of these four assays are presented in Figure 4. Consequently, these four assays (two hyper- and two hypo-methylated in brain tissue) constitute the proposed array of tests for the differential diagnosis of brain tissues.

**Figure 5:**
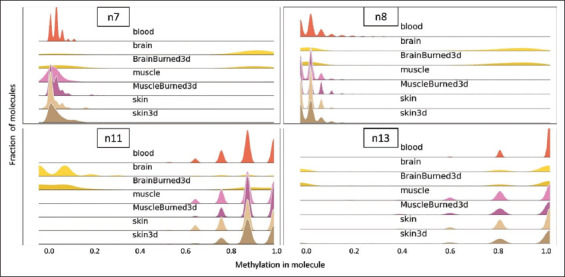
Distribution of reads by ranges of methylation for assays n7, n8, n11, and n13. 3d refers to air-dried for 3 days. Burned3d refers to burned over a flame and air-dried for 3 days.

A reasonable working assumption is that samples for diagnosis in cases related to this study may be found in a crime scene or a war zone for several days before sampling and might be in a poor state of preservation (e.g., after events such as shooting, explosion, or fire). Therefore, we decided to simulate these potential scenarios. To assess the effect of extreme field conditions on assay results, tissue samples were burned and air-dried outdoors for 3 days, and then underwent analysis. As are shown in Figure 5, the ability to identify the presence of brain tissue using the four diagnostic amplicons was maintained even under the challenging conditions tested. Assays n8, n11, and n13 preserved the sensitivity of discrimination between brain and non-brain tissue, while the n7 assay included a smaller fraction of brain tissue possessing a distinct methylation profile (Figure 5). This might slightly decrease the sensitivity of assay n7, while still producing accurate results. Employing strict thresholds, false positives are not expected under the conditions tested, while positive identification is expected to be achievable in all samples where the relative concentration of brain tissue is as low as 5%.

### 3.5. Mixed samples

**Figure 6:**
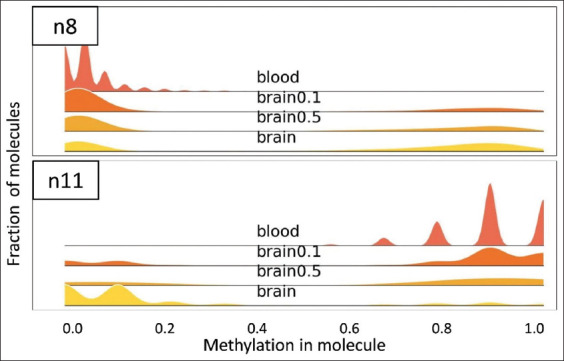
Distribution plot of reads by ranges of methylation for assays n8 and n11. These plots present the results for mixed samples: “brain0.5” mixed sample with 50% brain tissue, “brain0.1”—mixed sample with 10% brain tissue.

In a war zone or crime scene, a plausible scenario also involves the tissue of interest being mixed with other tissue types. To prove the feasibility of our approach in this scenario, we prepared two mixtures: one consisting of 50% brain tissue samples and 50% other tissues (heart, muscle, and skin), and another comprising 10% brain tissue samples and 90% other tissues (by weight). These mixtures were subsequently analyzed using our methodology. As demonstrated in regions n8 and n11 (Figure 6), our methodology allowed an identification in the mixed samples in both brain concentrations tested. Reasonably, the fraction of hypo- or hyper-methylated reads was correlated with the brain tissue concentration.

### 3.6. Final assay accuracy

The best diagnostic regions tested are proven to be accurate for strict methylation fraction thresholds: 0.1 for hypo-methylated regions and 0.9 for hyper-methylated ones. Requiring that 5% or more of the molecules in a sample fit these thresholds for sample identification allowed for complete accuracy for all eight confounding tissues tested, using each amplicon alone. This accuracy is also valid for degraded, burned, and air-dried samples. The identification is also demonstrated for samples containing as little as 10% brain content, and lower concentrations are also expected to be detected, assuming a random molecule subsection. It is also important to note that PCR and HTS error profiles are small enough to completely reject the possibility of technical errors for those thresholds.^27^

### 3.7. Bioinformatic approach

The challenge of accurate identification within mixed signals appears in many fields of science and forensics. Our approach for addressing this challenge in the context of methylation profile-based tissue identification leverages the complete information yielded in HTS. The effectiveness of the computational analysis is mostly owed to the consideration of each read as an information unit. A sample yields approximately 10^3^–5 × 10^6^ reads, each eventually represented by a methylation percentage value. In contrast, methylation assay results are typically summed and presented as the average level of methylation at each genomic position, up to dozens of values. Our approach allows the extraction of much more information from each sample, permitting the accurate identification of brain tissue presence within mixtures. Once we calculate the methylation percentage in each read, the decision threshold can be made with respect to the distribution of these values. For example, for assay n11, we see that most of the brain tissue reads are characterized by a methylation percentage lower than 20%; requiring that 50% of reads will be 20% methylated or lower will allow the accurate detection of brain in a pure sample. Requiring that 5% of reads exhibit 20% methylation or lower is expected to allow accurate detection in a sample composed of 10% brain. We avoid claims about further extrapolation of this to very low concentrations, because those are much less relevant for forensic application and can be affected by other limitations, such as sequencing accuracy and bisulfite conversion efficiency.

## 4. Discussion

The identification of tissue content in forensic samples is an important task, and the potential of methylation profiles for achieving this goal is recognized by recent studies. DNA methylation is proven to be stable, and its role in expression control makes it suitable for tissue identification.^10^,^28^ More specifically, the identification of brain tissue in samples may provide valuable information related to the condition of a missing person, and the presence of brain tissue in crime scenes or battlefields may allow, in combination with other evidence, to determine the death of a missing victim.

This study presents the development of a methylation-based assay for the identification of brain tissue and addresses practical concerns that are revealed by field condition exercises. Specifically, the topics of precision and reliability in field conditions are crucial to the potential application of such an assay, where an inaccuracy, especially a false positive result, may lead to detrimental consequences. The approach presented, which calls for HTS of bisulfite-treated DNA, has several advantages in terms of these accuracy concerns, stemming from the fact that the complete state of each molecule is known, rather than the average methylation rate in each position yielded by traditional methods such as specific restriction enzyme activity and real-time PCR probes. The final diagnostic array of four genomic loci selected in the present study showed high precision: for each, a methylation profile dominant in brain tissue was completely absent in all other tissues tested. For each, tens–hundreds of thousands of highly accurate sequence molecule reads were yielded when using the presented protocol, which was demonstrated to allow perfect detection accuracy in a tested sample containing as little as 10% brain. In theory, much lower brain content could also be detected.

While the tested tissue samples showed complete accuracy, various conditions may still cause false positive results. Untested tissues may exhibit methylation profiles identical to brain, unexpected sample contents or lab mistakes may hinder bisulfite activity, unspecific amplification in environmental samples may show sequence similarity, and more. Some controls offered mitigate such circumstances: four different genomic loci tested, each capable of accurate detection of the brain on its own, are unlikely to all give false results, and any contradiction should cause the rejection of the results. The correct amplification and bisulfite treatment were verifiable as both hyper-methylated and hypo-methylated tests were used, and a deviation in bisulfite activity levels can only cause a false positive result in one of these assay types. Another challenge related to the method is the successful amplification of bisulfite-treated DNA. Bisulfite treatment is harsh and may lead to DNA strand fragmentation. The resulting DNA is often shorter and of lower quality, making amplification less efficient for longer targets.

The results presented promise high-accuracy detection capabilities of brain tissue in forensic samples. However, additional work is needed before these assays can be used by forensic labs, including extensive validation iterations with larger sample sets, legal certification, and other relevant research. Nonetheless, this work presents an extensive application of HTS and bisulfite technologies for the application of tissue detection, dealing with issues such as mixed tissue samples, degraded samples, and high precision.

## 5. Conclusion

This study demonstrated the feasibility and reliability of using DNA methylation markers for the forensic identification of human brain tissue. By targeting highly specific genomic regions and leveraging HTS data, the developed assay provides accurate detection even in degraded or mixed tissue samples. These findings highlight the potential of epigenetic profiling as a powerful and stable molecular approach for tissue source determination, supporting its future integration into forensic practice.

## Data Availability

The data that support the findings of this study were deposited at the NCBI Sequence Read Archive (SRA) website (https://www.ncbi.nlm.nih.gov/sra); bioproject PRJNA1162678 (https://www.ncbi.nlm.nih.gov/bioproject/PRJNA1162678).

## Supplementary File

The score for each genomic position is calculated using the following Equation (S1):

where Mt_1_… Mt_k_ refers to *n* vectors in length *k*, describing the methylation state in *k* non-brain tissue samples in n genomic positions; Mb_1_…Mb_w_ refers to *n* vectors in length *k*, describing the methylation state in *w* brain tissue samples in n genomic positions; *p* is the penalty parameter; *b* is the “brain preference” parameter, representing the relative importance of methylation values in brain samples; and *d* is the ideal methylation state for each tissue, for each score type. The value of *d* is defined based on the sample type and the corresponding score classification. For brain samples, the hypo score was assigned a value of 0, while the hyper score was assigned 1. In contrast, for non-brain samples, the hypo score was assigned 1, and the hyper score was assigned 0.

Potential genomic regions containing at least five CpG sites within 300 bp were ranked based on CpG count and the mean informativeness score of the top 50% most informative sites using Equation (S2):

The parameters and their corresponding final values for Equation (S2) are defined as follows. The maximum length (max_len) was set to 300. A value of 1 was used to represent the bias toward the best position within the amplicon range (max_bias). The maximum amplicon size rewarded (max_rewarding_size) was set at 150. The bias toward amplicon length was given a weight of 0.0 (length_weight), while 0.5 was assigned to represent the fraction of top positions given higher importance (top_half_fraction).

For information regarding potential genomic position ranges, the sum of the best top_half_fraction positions was recorded as Top_half_sum. The score of the best position within the range was referred to as top_score, while the distance between the first and last CpG sites was defined as amplicon_len. In addition, the maximum and minimum scores within the range were denoted as max_score and min_score, respectively.
